# Graph regularized non-negative matrix factorization with prior knowledge consistency constraint for drug–target interactions prediction

**DOI:** 10.1186/s12859-022-05119-6

**Published:** 2022-12-29

**Authors:** Junjun Zhang, Minzhu Xie

**Affiliations:** 1 Key Laboratory of Computing and Stochastic Mathematics (LCSM) (Ministry of Education), School of Mathematics and Statistics, Hunan Normal University, Changsha, 410081 China; 2 College of Information Science and Engineering, Hunan Normal University, Changsha, 410081 China

**Keywords:** Graph regularized matrix factorization, Prior knowledge consistency constraint, Drug–target interaction prediction

## Abstract

**Background:**

Identifying drug–target interactions (DTIs) plays a key role in drug development. Traditional wet experiments to identify DTIs are expensive and time consuming. Effective computational methods to predict DTIs are useful to narrow the searching scope of potential drugs and speed up the process of drug discovery. There are a variety of non-negativity matrix factorization based methods to predict DTIs, but the convergence of the algorithms used in the matrix factorization are often overlooked and the results can be further improved.

**Results:**

In order to predict DTIs more accurately and quickly, we propose an alternating direction algorithm to solve graph regularized non-negative matrix factorization with prior knowledge consistency constraint (ADA-GRMFC). Based on known DTIs, drug chemical structures and target sequences, ADA-GRMFC at first constructs a DTI matrix, a drug similarity matrix and a target similarity matrix. Then DTI prediction is modeled as the non-negative factorization of the DTI matrix with graph dual regularization terms and a prior knowledge consistency constraint. The graph dual regularization terms are used to integrate the information from the drug similarity matrix and the target similarity matrix, and the prior knowledge consistency constraint is used to ensure the matrix decomposition result should be consistent with the prior knowledge of known DTIs. Finally, an alternating direction algorithm is used to solve the matrix factorization. Furthermore, we prove that the algorithm can converge to a stationary point. Extensive experimental results of 10-fold cross-validation show that ADA-GRMFC has better performance than other state-of-the-art methods. In the case study, ADA-GRMFC is also used to predict the targets interacting with the drug olanzapine, and all of the 10 highest-scoring targets have been accurately predicted. In predicting drug interactions with target estrogen receptors alpha, 17 of the 20 highest-scoring drugs have been validated.

## Background

According to the DrugBank database, there are over 500,000 drugs for different diseases. However, there are still many diseases for which we have no effective drugs, and there is a long way for drug discovery and drug repurposing. The process of drug discovery and drug repurposing takes some important steps such as finding valid target proteins and discovering proper chemical compounds to interact with the targets (i.e. identifying drug–target interactions or binding affinity between drugs and proteins) [1]. Determining drug–target interactions (DTIs) via wet experiments is both time-consuming and expensive [2, 3]. To increase the probabilities of discovering new drugs or new applications of approved drugs, accurate computational methods to predict DTI are in urgent need to choose a small number of compounds for the wet experiments.

Based on the crystal structure of the target binding site, Cheng et al. [4] constructed maximal affinity model using docking simulations and calculated the maximal affinity values associated with the drug. Campillos et al. [5] used side-effect similarity of drugs to infer the probability of two drug sharing a target. The above two methods could not predict DTIs for targets without known crystal structure or drugs without known side-effects.

To effectively predict DTIs in large scale, a lot of computation models have been introduced. For example, Yamanishi et al. [6] computed drug chemical structure similarities, amino acid sequence similarities of target proteins, and proposed a bipartite graph learning method to predict DTIs by eigenvalue decomposition based on and known drug–target interactions. Based on the same drug structure similarities, sequence similarities of proteins and known drug–target interactions, Bleakley and Yamanishi [7] proposed bipartite local models (BLM) to predict target proteins for a given drug, and the drugs targeting a given protein. Laarhoven et al. [8] proposed Gaussian interaction profile (GIP) kernel method to calculate the similarities between targets (and drugs), and regularized least squares (RLS) was used to predict unknown DTIs. The Kronecker RLS model [9] was proposed based on RLS. However, these methods can not predict DTIs for the drugs or targets if they have no known interactions. In order to compensate the lack of interaction information, based on GIP, Laarhoven and Marchiori [10] proposed a weighted nearest neighbor method to predict DTIs. Mei et al. [11] derived training data from neighbors of new drug (target) candidates, and proposed BLM-NII method by integrating neighbor-based interaction-profile inferring into BLM for DTI predictions.

Instead of utilizing the attributes of drug chemical structure and sequence of target proteins separately, more and more methods integrated multiple features and then built classifiers to make prediction. Wang and Zeng [12] transformed the DTI prediction problem into a two-layer graphical model, used restricted Boltzmann machine to predict diverse types of DTIs, such as direct and indirect interactions. Based on multiple information, random walk with restarts (RWR) was used for DTIs feature extraction on positive-unlabeled learning method [13] and DTINet method [14]. In order to predict large scale DTIs, convolutional neural network (CNN) was applied to to extract drug and target features information [15–18]. FCS [19] and KGE_NFM [20] were proposed to obtain the low-rank representations for information of multi-omics in DTI prediction. Other information fusion methods including negative sample screening framework [21], SITAR framework [22] and multiple kernels learning [23, 24] were successively proposed. However, the computation complexity of large scale similarity matrices is high.

The known DTIs are usually represented by a drug–target interaction matrix, and matrix factorization method has been widely used in DTI prediction. Matrix factorization is a embedding model that is used to decompose interaction matrix into two feature matrices of low ranks which represents the interactions between drugs and targets as the product of feature matrices. For example, Gönen [25] proposed a kernelized Bayesian matrix factorization with twin kernels method to predict DTIs. The twin kernel matrices were constructed by chemical similarity function of drug compounds and genomic similarity function of target proteins. Combined logistic matrix decomposition with neighborhood regularization [26] and a variational Bayesian multiple kernel logistic matrix factorization method [27] were proposed to infer interactions. MSCMF [28], GRMF [29], $L_{2,1}$-GRMF [30], GRGMF [31], HCNMF [32], CHNMF [33] and SRCMF [34] were proposed by means of graph regularization. However, their performances are still not satisfying due to the lost information of these decompose strategies.

To implement matrix factorization, the above methods used either the alternating least squares algorithm [35] or the multiplicative update algorithm [36]. However, there are other efficient algorithms to implement matrix factorization. Hoyer [37] introduced a sparseness measure, and proposed a projected gradient descent algorithm for non-negative matrix factorization with sparseness constraints. Lin [38] proposed an improved projected gradient method for NMF with bound constrains. It has not been proved that the alternating least squares algorithm and the multiplicative update algorithm converge to a stationary point when convergence does occur [39]. Due to the use of a simple geometric rule for the step size, gradient descent methods are very sensitive to the initialization [40] and often produce a poor factorization. To overcome the limitations of the above methods, the alternating direction method (ADM) has attracted attention. To obtain a higher-quality matrix factorization with less computing time, Zhang [39] extended the alternating direction method (ADM) to solve NMF. Based on the above algorithm, Xu et al. [41] devised an improved alternating direction algorithm (ADA) to solve the non-negative matrix factorization-and-completion problem.

The high-dimensional data are in fact sampled from a nonlinear low-dimensional manifold embedded in the high-dimensional space, and according to [42], the model learning performance can be greatly improved if the intrinsic geometrical structure of the manifold have been taken into account. Shang et al. [43] showed that a graph dual regularization helps NMF improve clustering performance since the graph dual regularization considers the underlying geometric structures of both the data manifold and the feature manifold. In this paper, in order to predict DTIs more accurately and quickly, we propose an Alternating Direction Algorithm to solve Graph Regularized non-negative Matrix Factorization with prior knowledge consistency constraint (ADA-GRMFC). The prior knowledge consistency constraint aims to ensure that the decomposition result is consistent with the prior knowledge of known DTIs. The alternating direction algorithm ensures that ADA-GRMFC can converge to KKT point.

Extensive experimental results show that ADA-GRMFC has better performance than other state-of-the-art methods. In case studies involving the drug olanzapine and the target estrogen receptor alpha, all the 10 highest-scoring targets predicted to interact with olanzapine, and 17 of the 20 highest-scoring drugs predicted to interact with estrogen receptor alpha have been validated by wet experiments. The case studies show that, for drugs that do not have any known target proteins and for proteins that are so far not approved as drug targets, ADA-GRMFC also has good prediction performance.

## Materials

The experimental data include known drug–target interactions, drug chemical structures and target protein sequences. They were from public databases BRENDA [44], KEGG BRITE [45], SuperTarget [46] and DrugBank [47] and were downloaded from the website: http://web.kuicr.kyoto-u.ac.jp/supp/yoshi/drugtarget/.

The target proteins have the following four types: nuclear receptors (NR), G protein-coupled receptors (GPCR), ion channels (IC) and enzymes (E). Accordingly, the benchmark known drug–target interactions are divided into four datasets NR, GPCR, IC and E. The sizes of the four datasets are different. In the NR dataset, there are 90 known interactions between 54 drugs and 26 nuclear receptors; in the GPCR dataset, there are 635 known interactions between 223 drugs and 95 G protein-coupled receptors; in the IC dataset, there are 1476 known interactions between 210 drugs and 204 ion channels; and in the E dataset, there are 2926 known interactions between 445 drugs and 664 enzymes. The known interactions between *n* drugs and *m* proteins are recorded by an $n \times m$ DTI matrix *Z*. If the *i*th drug is approved to target the *j*th protein, $Z_{i,j}=1$; otherwise $Z_{i,j}=0$. The information of the four datasets are shown in Table 1.

**Table 1:** The information of the benchmark datasets

Datasets	NR	GPCR	IC	E
Interactions	90	635	1476	2926
Drugs	54	223	210	445
Targets	26	95	204	664
Sparseness (%)	93.59	97.00	96.55	99.01

The structural similarities between drugs were calculated using SIMCOMP [48] according to the size of the common substructures between two drugs.

An $n\times n$ matrix $S^d$ is used to record the similarity information between *n* drugs. The sequence similarity of the target proteins used the normalized version of the Smith-Waterman score [49]. Let $p_1$ and $p_2$ represent two proteins and *SW*(., .) be the original Smith-Waterman alignment score. The Smith-Waterman score of the normalized version of $p_1$ and $p_2$ is $s({p_1},{p_2}) = \frac{{SW({p_1},{p_2})}}{{\sqrt{SW({p_1},{p_1})} \sqrt{SW({p_2},{p_2})} }}$. An $m\times m$ matrix $S^t$ is used to store the similarity information between *m* target proteins.

## Methods

After construction of the DTI matrix *Z*, the drug similarity matrix $S^d$ and a target similarity matrix $S^t$, DTI prediction is transformed into non-negative factorization of the DTI matrix with graph dual regularization terms and a prior knowledge consistency constraint. The graph dual regularization terms are used to integrate the information from the drug similarity matrix and the target similarity matrix, in order to take the intrinsic geometrical structures of the related manifolds into account. Finally, an alternating direction algorithm is used to solve the matrix factorization. Furthermore, we prove the convergence of the algorithm.

### Non-negative matrix factorization

In order to obtain low-dimensional feature representations of drugs and targets in the drug–target interaction space, factorization of the DTI matrix is widely adopted. The general form of the matrix factorization is as follows:

1 $$\begin{aligned} Z\approx X Y^T, \end{aligned}$$

where *X* and *Y* are the latent feature matrices of drugs and targets respectively, $X \in {R^{n \times k}}, Y \in {R^{m \times k}}$, and *k* is the rank of feature vectors of drugs and targets ($k \ll \min (m,n)$).

To improve interpretability, the non-negativity constraint of *X* and *Y* is usually added. The optimization model of non-negative matrix factorization (NMF) is as follows:

2 $$\begin{array}{*{20}l} {\min \left\| {Z - XY^{T} } \right\|_{F}^{2} } \\ {s.t.~X \ge 0,Y \ge 0.} \\ \end{array}$$

### Graph regularized non-negative matrix factorization

NMF aims to well approximate the DTI matrix by finding two low rank matrices, but fail to consider the geometric information in the original data. To integrate the geometric information, Cai et al. [50] proposed graph regularized non-negative matrix factorization (GNMF) which introduces a graph regularization item. The cost function of GNMF is as follows:

3 $$\begin{aligned} \mathcal {O}_{gr}=\frac{1}{2}\left\| {Z - X{Y^T}} \right\| _F^2&+{\lambda }{\text {Tr}}({Y^T}(Y^T(D-W)) Y)\\&s.t. ~ X \ge 0, Y \ge 0, \end{aligned}$$

where ${\text {Tr}}$ is the trace of a matrix, $\lambda$ is regularization parameter, *W* is the weight matrix representing a neighbor graph of the data points, and *D* is a diagonal matrix such that ${D_{ii}} = \sum \nolimits _l {{W_{il}}}$. The matrix $D-W$ is called graph Laplacian and denoted by $\mathcal {L}$ in the following. Furthermore, considering the geometric structure of data manifold and feature manifold, Shang et al. [43] proposed graph dual regularization non-negative matrix factorization (GDNMF), whose cost function is:

4 $$\begin{aligned} \begin{aligned} \mathcal {O}_{gd}&=\frac{1}{2}\left\| {Z - X{Y^T}} \right\| _F^2 +{\lambda _y}{\text {Tr}}({Y^T}(Y^T \mathcal {L}_y Y)\\&+{\lambda _x}{\text {Tr}}({X^T}(X^T \mathcal {L}_x X)\\&s.t. ~ X \ge 0, Y \ge 0. \end{aligned} \end{aligned}$$

From the similarity matrices $S^d$ and $S^t$, we could obtain the geometric information of drugs and targets. First we construct two *p*-nearest neighbor graphs $N^d$ and $N^t$ of drugs and targets, respectively.

For two drugs $d_i$ and $d_j$, the weight of the edge between vertices *i* and *j* in the *p*-nearest neighbor graph $N^d$ is defined as follows.

5 $$\begin{aligned} {N_{ij}^d} = \left\{ \begin{array}{l} 1, \quad j \in {\mathcal {N}_p}(i) \text { and } i \in {\mathcal {N}_p}(j) \\ 0, \quad j \notin {\mathcal {N}_p}(i) \text { and } i \notin {\mathcal {N}_p}(j) \\ 0.5, \quad \text {otherwise,} \\ \end{array} \right. \end{aligned}$$

where $\mathcal {N}_p(i)$ and $\mathcal {N}_p(j)$ are the sets of *p* most similar drugs of drugs $d_i$ and $d_j$ according to $S^d$, respectively. $N^d$ is used to make the drug similarity matrix $S^d$ sparse as follows.

6 $$\begin{aligned} {\hat{S}}_{ij}^d = {N_{ij}^d}S_{ij}^d, \forall i, j. \end{aligned}$$

${\hat{S}}^d$ is used as the weight matrix representing the drug neighbor graph. The graph Laplacian of ${\hat{S}}^d$ is ${\mathcal {L}_d} = {D^d} - {{\hat{S}}^d}$, where $D^d$ is a diagonal degree matrix with $D_{ii}^d = \sum \limits _r {{\hat{S}}_{ir}^d}$.

The same processing is performed on the target similarity matrix $S^t$ and we calculated out ${\hat{S}}^t$ the weight matrix representing the target neighbor graph as follows.

7 $$\begin{aligned} {\hat{S}}_{ij}^t = {N_{ij}^t}S_{ij}^t, \forall i, j. \end{aligned}$$

The graph Laplacian of ${\hat{S}}^t$ is ${\mathcal {L}_t} = {D^t} - {{\hat{S}}^t}$, where $D^t$ is diagonal degree matrix, $D_{jj}^t = \sum \limits _q {{\hat{S}}_{jq}^t}$.

Since the normalized graph Laplacian usually performs better in many actual applications, we adopted the following normalized graph Laplacian forms of $\mathcal {L}_d$ and $\mathcal {L}_t$.

8 $$\begin{aligned}{} & {} \begin{aligned} {\widetilde{{\mathcal {L}}}_d} = {\left( {{D^d}} \right) ^{ - 1/2}}{{{\mathcal {L}}}_d}{\left( {{D^d}} \right) ^{ - 1/2}}, \end{aligned} \end{aligned}$$

9 $$\begin{aligned}{} & {} \begin{aligned} {\widetilde{{\mathcal {L}}}_t} = {\left( {{D^t}} \right) ^{ - 1/2}}{{{\mathcal {L}}}_t}{\left( {{D^t}} \right) ^{ - 1/2}}. \end{aligned} \end{aligned}$$

The graph dual regularization non-negative matrix factorization (GDNMF) for DTI prediction problem is formulated as follows.

10 $$\begin{aligned} \begin{aligned} \mathop {\min }\limits _{(X,Y,Z)}&\frac{1}{2}\left\| {Z - X{Y^T}} \right\| _F^2 +{\lambda _d}{\text {Tr}}({X^T}\widetilde{\mathcal {L}}_d X)\\&+ {\lambda _t}{\text {Tr}}({Y^T}\widetilde{\mathcal {L}}_t Y).\\ s.t. ~&X \ge 0, Y \ge 0, \\ \end{aligned} \end{aligned}$$

where $\lambda _d$ and $\lambda _t$ are regularization parameters.

### Graph dual regularized non-negative matrix factorization with prior knowledge constraint for DTI prediction

To ensure the matrix decomposition result is consistent with the prior knowledge of known DTIs, we introduce a Prior knowledge Constraint in GDNMF and formulate the DTI prediction problem as the following optimization problem (abbreviated as GRMFC).

11 $$\begin{aligned} \begin{aligned} \mathop {\min }\limits _{(X,Y,Z)}&\frac{1}{2}\left\| {Z - X{Y^T}} \right\| _F^2 +{\lambda _d}{\text {Tr}}({X^T}\widetilde{\mathcal {L}}_d X)\\&+ {\lambda _t}{\text {Tr}}({Y^T}\widetilde{\mathcal {L}}_t Y).\\ s.t. ~&X \ge 0, Y \ge 0, \\&{\mathrm{\mathscr {P}}_\Omega }(Z - XY^T) = 0,\\ \end{aligned} \end{aligned}$$

where $\Omega$ indexes the known drug–target interactions, *i.e.* the elements whose values are 1 in *Z*. ${\mathrm{\mathscr {P}}_\Omega }(S)$ returns a copy of *S* that zeros out the elements whose indices are not in $\Omega$, which is defined as follows.

$$\begin{aligned} {{\mathscr {P}_\Omega }(S)_{ij}} = \left\{ \begin{array}{l} {S_{ij}},(i,j) \in \Omega , \\ 0,(i,j) \notin \Omega . \\ \end{array} \right. \end{aligned}$$

Since the elements whose values are 1 in *Z* represent the known validated drug–target interactions, and we introduce the prior knowledge consistency constraint ${\mathrm{\mathscr {P}}_\Omega }(Z - XY^T) = 0$ to ensure that the matrix fraction $XY^T$ remains the known DTIs and does not lose the prior knowledge.

GRMFC is a non-convex optimization problem, and it is difficult to obtain its accurate solution. Inspired by the iteration algorithm in [41], we used an adapted alternating direction algorithm to obtain a local optimal solution. The alternating direction algorithm (ADA) is an iteration algorithm that alternatively updates *X* and *Y*. In order to use ADA to solve GRMFC efficiently, we introduce auxiliary matrices *M*, *U* and *V*, and transform (11) into the following equivalent form.

12 $$\begin{aligned} \begin{aligned} \mathop {\min }\limits _{(U,V,X,Y,M)}&\frac{1}{2}\left\| {M - X{Y^T}} \right\| _F^2 +{\lambda _d}{\text {Tr}}({X^T}\widetilde{\mathcal {L}}_d X)\\&+ {\lambda _t}{\text {Tr}}({Y^T}\widetilde{\mathcal {L}}_t Y).\\ s.t. ~&X = U, {Y^T} = V, \\&U \ge 0, V \ge 0, \\&{\mathrm{\mathscr {P}}_\Omega }(Z - M) = 0,\\ \end{aligned} \end{aligned}$$

where $U \in {R^{n \times k}}$, $V \in {R^{k \times m}}$. The auxiliary matrix *M* is regarded as the predicted drug–target interaction matrix.

The augmented Lagrangian of (12) is:

13 $$\begin{aligned} &{\mathscr {L}}(X,Y,M,U,V,\Lambda ,\Pi )\\&= \frac{1}{2}\left\| {M - X{Y^T}} \right\| _F^2+ {\lambda _d}{\text {Tr}}({X^T}{\widetilde{\mathcal {L}}}_d X) \\&+{\lambda _t}{\text {Tr}}({Y^T}{\widetilde{\mathcal {L}}}_t Y)+\Lambda \bullet (X - U)\\&+ \Pi \bullet (Y^T - V)+ \frac{\alpha }{2}\left\| {X - U} \right\| _F^2\\&+\frac{\beta }{2}\left\| {Y^T - V} \right\| _F^2, \end{aligned}$$

where $\Lambda$ and $\Pi$ are Lagrangian multipliers, $\Lambda \in {R^{n \times k}}$, $\Pi \in {R^{k \times m}}$, and $\alpha , \beta > 0$ are penalty parameters.

The alternating direction algorithm successively updates the values of matrices *X*, *Y*, *M*, *U* and *V* one at a time, such that $\mathscr {L}$ reaches the minimum with respect to the updated matrix while the other matrices take their most recent values. The updating rules are as follows.

$$\begin{aligned} X_{{i + 1}} & = \mathop {\arg \min }\limits_{X} {\mathscr{L}}(X,Y_{i} ,M_{i} ,U_{i} ,V_{i} ,\Lambda _{i} ,\Pi _{i} ), \\ Y_{{i + 1}} & = \mathop {\arg \min }\limits_{Y} {\mathscr{L}}(X_{{i + 1}} ,Y,M_{i} ,U_{i} ,V_{i} ,\Lambda _{i} ,\Pi _{i} ), \\ M_{{i + 1}} & = \mathop {\arg \min }\limits_{{{\mathscr{P}}_{\Omega } (Z - M) = 0}} {\mathscr{L}}(X_{{i + 1}} ,Y_{{i + 1}} ,M,U_{i} ,V_{i} ,\Lambda _{i} ,\Pi _{i} ), \\ U_{{i + 1}} & = \mathop {\arg \min }\limits_{{U \ge 0}} {\mathscr{L}}(X_{{i + 1}} ,Y_{{i + 1}} ,M_{{i + 1}} ,U,V_{i} ,\Lambda _{i} ,\Pi _{i} ), \\ V_{{i + 1}} & = \mathop {\arg \min }\limits_{{V \ge 0}} {\mathscr{L}}(X_{{i + 1}} ,Y_{{i + 1}} ,M_{{i + 1}} ,U_{{i + 1}} ,V,\Lambda _{i} ,\Pi _{i} ), \\ \Lambda _{{i + 1}} & = \Lambda _{i} + \gamma \alpha (X_{{i + 1}} - U_{{i + 1}} ), \\ \Pi _{{i + 1}} & = \Pi _{i} + \gamma \beta (Y_{{i + 1}} - V_{{i + 1}} ). \\ \end{aligned}$$

In closed form, the updating rules are as follows.

14 $$\begin{aligned} &{X_{i + 1}} = ({M_i}{Y_i} - {\lambda _d}{\widetilde{{\mathcal {L}}}_d}X + \alpha {U_i}- {\Lambda _i}) \\&\quad \quad {(Y_i^T{Y_i} + \alpha I)^{ - 1}},\\&{Y_{i + 1}} = ({M_{i}^T}{X_{i+1}} - {\lambda _t}{\widetilde{{\mathcal {L}}}_t}Y + \beta {V_i} - {\Pi _i})\\&\quad \quad {(X_{i+1}^T{X_{i+1}} + \beta I)^{ - 1}},\\&{M_{i + 1}} = X_{i + 1}Y_{i + 1}^T+{\mathscr {P}}_{\Omega }(M-X_{i + 1}Y_{i + 1}^T),\\&{U_{i + 1}} ={\mathscr {P}}_{+}(X_{i + 1}+{\Lambda _i}/ \alpha ),\\&{V_{i + 1}} = {\mathscr {P}}_{+}(Y_{i + 1}+{\Pi _i}/ \beta ),\\&{\Lambda _{i + 1}} = {\Lambda _i} + \gamma \alpha ({X_{i + 1}} - {U_{i + 1}}), \\&{\Pi _{i + 1}} = {\Pi _i} + \gamma \beta ({Y_{i + 1}} - {V_{i + 1}}), \end{aligned}$$

where $(\mathscr {P}_{+}(S))_{ij}=max\{S_{ij}, 0\}$, and $\gamma$ is a step length parameter which is set as 1.618 in the following experiments according to [41].

The iteration process will terminated when the changes of *M* smaller than a given smaller threshold. The pseudocode of the algorithm (ADA-GRMFC) is shown in Algorithm 1. The proof of convergence of ADA-GRMFC is shown in Additional file 1: Appendix.
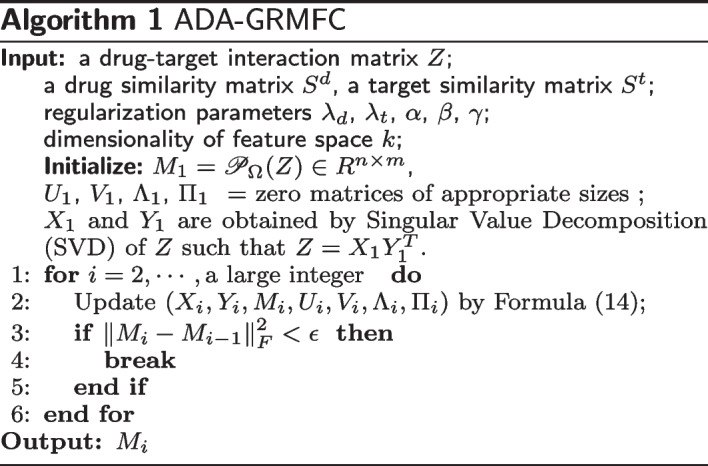


## Experiments

The performance of ADA-GRMFC has been tested in the following three aspects. First, ADA-GRMFC was compared with other state-of-the-art algorithms using the four datasets: NR, GPCR, IC and E. Second, we analyzed the effect of graph dual regularization terms on the prediction accuracy of ADA-GRMFC using ablation experiments. Third, we tested ADA-GRMFC using case studies.

To compare performances of DTI prediction algorithms, 5 repetitions of 10-fold cross-validation were performed. The final test results were the averages of the 5 repetitions of 10-fold cross-validation. The cross-validation experiments are conducted under the following two scenarios [9].

1. $CV_d$: The drugs are divided in ten subsets (folds), each fold is selected in turn as the test dataset and the other remained 9 folds are used as the training dataset. If the *i*th drug is in the test dataset, the elements in the *i*th row of *Z* are all set 0, which means the known interactions with tested drugs are removed from the input DTI matrix. It aims to evaluate the targeted protein prediction performance for the drugs without any known interactive targets.
2. $CV_t$: The targets are divided in ten subsets (folds), each fold is selected in turn as the test dataset and the other remained 9 folds are used as the training dataset. If the *j*th target in the test dataset, the elements in the *j*th column of *Z* are all set 0, which means the known interactions with tested targets are removed from the input DTI matrix. It aims to evaluate the targeting drug prediction performance for the targets without any known interactive drugs.

The area under receiver operating characteristic curve (AUC) and area under the precision-recall curve (AUPR) are used as performance evaluation metrics in the following experiments.

### Comparison with state-of-the-art methods

To demonstrate the effectiveness of ADA-GRMFC in predicting DTIs, we compared ADA-GRMFC with the following seven methods, namely BLM-NII [11], WKNKN [10], RLS-WNN [8], GRMF [29], WGRMF, CMF [28], SRCMF [34], where WGRMF is a weighted form of GRMF.

#### Parameter settings

For BLM-NII, the combination weight $\alpha =0.5$. For WKNKN, the parameters $K=5$, $\eta =0.7$. According to the original literature of GRMF, WGRMF, CMF and SRCMF, some parameters are automated chosen using grid search [51] based on the AUPR value. Based on a previous research [29], rank *k* of the matrices *X* and *Y* was selected from $\left\{ {50, 100} \right\}$. For GRMF, WGRMF, CMF and SRCMF, the regularization parameter $\lambda _l$ was selected from $\{2^{-2}, 2^{-1}, 2^{0}, 2^{1}\}$. For GRMF, WGRMF and ADA-GRMFC, $\lambda _d$ and $\lambda _t$ were selected from $\{0, 10^{-4}, 10^{-3}, 10^{-2}, 10^{-1}\}$. For CMF and SRCMF, $\lambda _d$ and $\lambda _t$ were selected from {$2^{-3}, 2^{-2}, 2^{-1}, 2^{0}, 2^{1}, 2^{2}, 2^{3}, 2^{4}, 2^{5}$}.

**Fig. 1:**
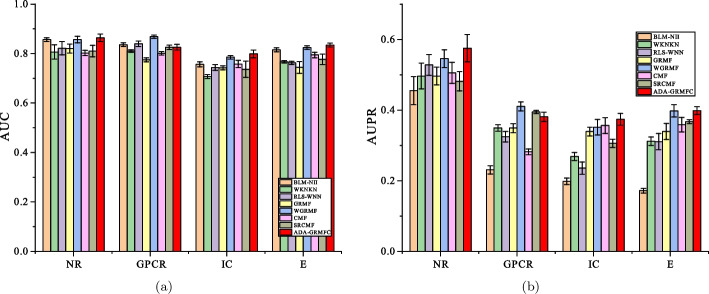
AUC values and AUPR values of the methods on the four datasets under $CV_d$. **a** Histogram with error bars of AUC. **b** Histogram with error bars of AUPR

**Fig. 2:**
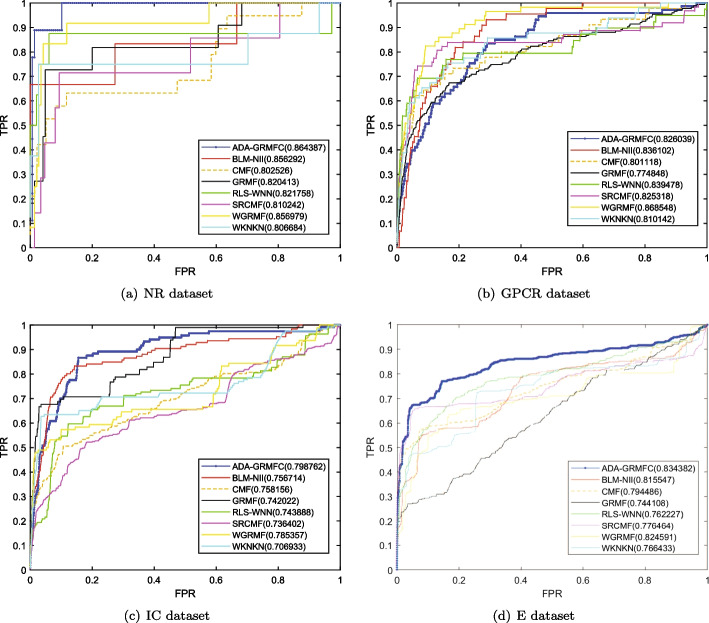
ROC curves for different methods are plotted together under CVd on NR dataset, GPCR dataset, IC dataset, E dataset, respectively

**Fig. 3:**
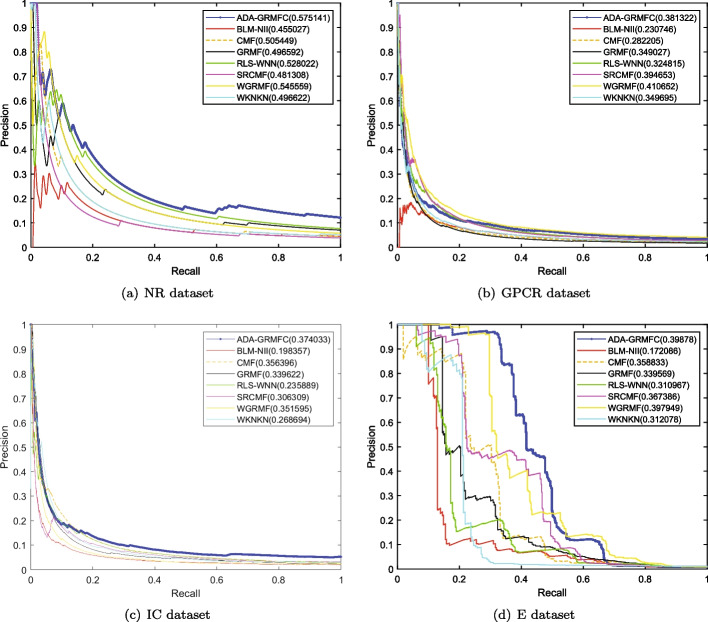
PR curves for different methods are plotted together under $CV_d$ on NR dataset, GPCR dataset, IC dataset, E dataset, respectively

In terms of ADA-GRMFC, the optimal parameters combination of $\alpha$, $\beta$ and $\gamma$ are different under different experiment scenario, which makes the parameter settings more complicated. When tuning the parameter $\alpha$, we set other parameters as their optimal values. The same settings were applied to $\beta$ and $\gamma$. Figures 1, 2 and 3 in the appendix file showed the impact of the parameter $\alpha$, $\beta$ and $\gamma$ on AUC and AUPR, respectively. When the parameters change, the performances of ADA-GRMFC varied more significantly on NR datasets and GPCR datasets than on IC datasets and E datasets. The results show that the best performance was achieved when $\alpha = 0.5$, $\beta =0.01$ and $\gamma =1.618$. Thus, in the following experiments, for ADA-GRMFC, $\alpha =0.5$, $\beta =0.01$, $\gamma = 1.618$. The parameters $\epsilon =10^{-6}$.

#### Prediction results

Under the $CV_d$ scenario, ADA-GRMFC performs better than other methods in terms of AUC and AUPR on NR, IC, and E datasets. The AUC values of ADA-GRMFC are 0.860748, 0.798762, and 0.834382 on NR, IC, and E datasets, respectively. The AUPR values of ADA-GRMFC are 0.574956, 0.374033, and 0.39878 on NR, IC, and E datasets, respectively. On the GPCR dataset, WGRMF achieve the highest AUC and AUPR values, which are 0.868548 and 0.410652, respectively. The AUC and AUPR values of ADA-GRMFC are higher than those of other algorithms except WGRMF. The AUC and AUPR values of the different algorithms on the four datasets are shown in Tables 2 and 3, respectively. The AUC and AUPR histograms with error bars of different algorithms are shown in Figure 1a, b, respectively. The receiver operating characteristic (ROC) curves and the precision-recall (PR) curves of different methods on the four datasets are shown in Figs. 2 and 3, respectively.

**Table 2:** AUC values of different algorithms under $CV_d$ scenario

Method	NR	GPCR	IC	E
BLM-NII	0.856292 (0.0077)	0.836102 (0.0073)	0.756714 (0.0102)	0.815547 (0.0080)
WKNKN	0.806684 (0.0289)	0.810142 (0.0048)	0.706933 (0.0079)	0.766433 (0.0050)
RLS-WNN	0.821758 (0.0273)	0.839478 (0.0116)	0.743888 (0.0113)	0.762227 (0.0066)
GRMF	0.820413 (0.0185)	0.774848 (0.0082)	0.742022 (0.0080)	0.744108 (0.0240)
WGRMF	0.856979 (0.0135)	**0.868548** (0.0065)	0.785357 (0.0070)	0.824591 (0.0071)
CMF	0.802526 (0.0109)	0.801118 (0.0069)	0.758156 (0.0144)	0.794486 (0.0109)
SRCMF	0.810242 (0.0227)	0.825318 (0.0093)	0.736402 (0.0329)	0.776464 (0.0214)
ADA-GRMFC	**0.864387** (0.0153)	0.826039 (0.0119)	**0.798762** (0.0158)	** 0.834382** (0.0082)

The maximum AUC on each dataset is shown in bold. Standard deviation is shown in parentheses

**Table 3:** AUPR values of different algorithms under $CV_d$ scenario

Method	NR	GPCR	IC	E
BLM-NII	0.455027 (0.0395)	0.230746 (0.0118)	0.198357 (0.0091)	0.172086 (0.0068)
WKNKN	0.496622 (0.0366)	0.349695 (0.0096)	0.268694 (0.0113)	0.312078 (0.0121)
RLS-WNN	0.528022 (0.0294)	0.324815 (0.0149)	0.235889 (0.0176)	0.310967 (0.0232)
GRMF	0.496592 (0.0252)	0.349027 (0.0129)	0.339622 (0.0124)	0.339569 (0.0227)
WGRMF	0.545559 (0.0252)	**0.410652** (0.0126)	0.351595 (0.0223)	0.397949 (0.0176)
CMF	0.505449 (0.0299)	0.282205 (0.0081)	0.356396 (0.0227)	0.358833 (0.0205)
SRCMF	0.481308 (0.0273)	0.394653 (0.0049)	0.306309 (0.0116)	0.367386 (0.0054)
ADA-GRMFC	**0.575141** (0.0388)	0.381322 (0.0130)	**0.374033** (0.0165)	**0.39878** (0.0112)

The maximum AUPR on each dataset is shown in bold. Standard deviation is shown in parentheses

**Fig. 4:**
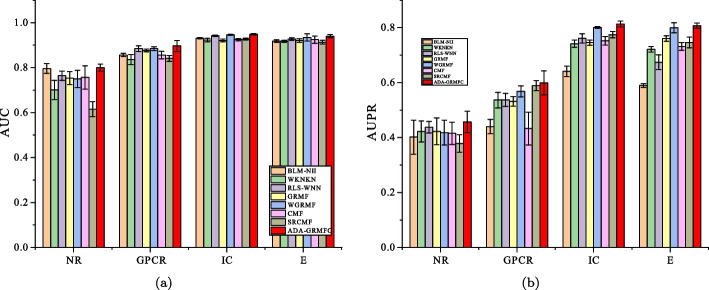
AUC values and AUPR values of the methods on the four datasets under $CV_t$. **a** Histogram with error bars of AUC. **b** Histogram with error bars of AUPR

**Fig. 5:**
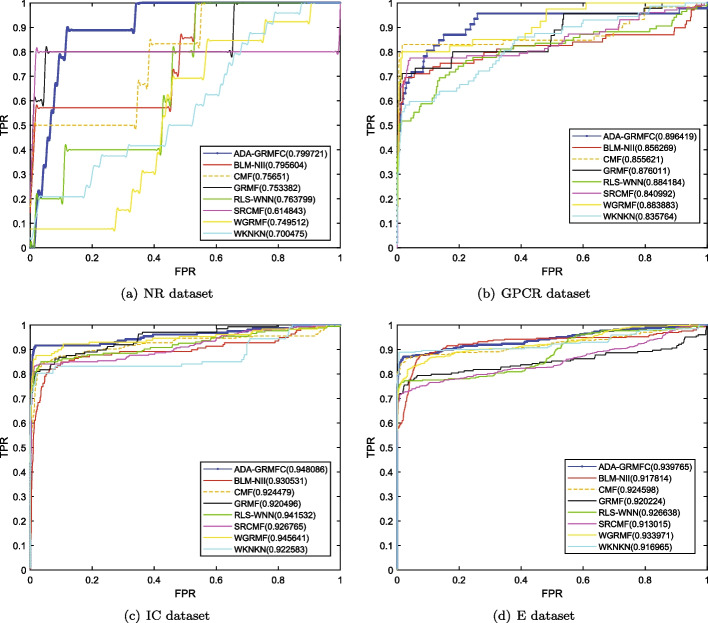
ROC curves for different methods are plotted together under $CV_t$ on NR dataset, GPCR dataset, IC dataset, E dataset, respectively

**Fig. 6:**
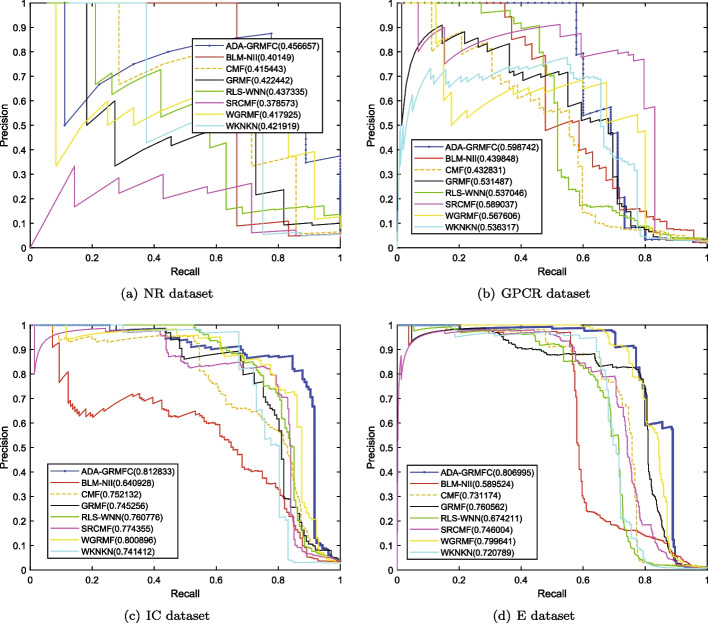
PR curves for different methods are plotted together under $CV_t$ on NR dataset, GPCR dataset, IC dataset, E dataset, respectively

Under the $CV_t$ scenario, the AUC and AUPR values of ADA-GRMFC are higher than the other methods on the four datasets. The AUC values of ADA-GRMFC are 0.799721, 0.896419, 0.948086, and 0.939765 on NR, GPCR, IC, and E datasets, respectively. The AUPR values of ADA-GRMFC on NR, GPCR, IC, and E datasets are 0.454528, 0.598742, 0.812833, and 0.806995, respectively. The AUC values and AUPR values of different algorithms on the four datasets are shown in Tables 4 and 5, respectively. The AUC and AUPR histograms with error bars of different algorithms are shown in Fig. 4a, b, respectively. ROC and PR curves of different algorithms are shown in Figs. 5 and 6 on the four datasets, respectively.

**Table 4:** AUC values of different algorithms under $CV_t$ scenario

Method	NR	GPCR	IC	E
BLM-NII	0.795604 (0.0217)	0.856269 (0.0071)	0.930531 (0.0029)	0.917814 (0.0056)
WKNKN	0.700475 (0.0430)	0.835764 (0.0217)	0.922583 (0.0079)	0.916965 (0.0042)
RLS-WNN	0.763799 (0.0208)	0.884184 (0.0128)	0.941532 (0.0031)	0.926638 (0.0053)
GRMF	0.753382 (0.0293)	0.876011 (0.0063)	0.920496 (0.0060)	0.920224 (0.0074)
WGRMF	0.749512 (0.0384)	0.883883 (0.0083)	0.945641 (0.0024)	0.933971 (0.0161)
CMF	0.75651 (0.0520)	0.855621 (0.0164)	0.924479 (0.0051)	0.924598 (0.0161)
SRCMF	0.614843 (0.0333)	0.840992 (0.0127)	0.926765 (0.0049)	0.913015 (0.0082)
ADA-GRMFC	**0.799721** (0.0154)	**0.896419** (0.0245)	**0.948086** (0.0038)	**0.939765** (0.0070)

The maximum AUC on each dataset is shown in bold. Standard deviation is shown in parentheses

**Table 5:** AUPR values of different algorithms under $CV_t$ scenario

Method	NR	GPCR	IC	E
BLM-NII	0.40149 (0.0618)	0.439848 (0.0259)	0.640928 (0.0191)	0.589524 (0.0069)
WKNKN	0.421919 (0.0382)	0.536317 (0.0281)	0.741412 (0.0131)	0.720789 (0.0100)
RLS-WNN	0.437335 (0.0206)	0.537046 (0.0235)	0.760776 (0.0169)	0.674211 (0.0266)
GRMF	0.422442 (0.0486)	0.531487 (0.0175)	0.745256 (0.0091)	0.760562 (0.0100)
WGRMF	0.417925 (0.0447)	0.567606 (0.0201)	0.800896 (0.0036)	0.799641 (0.0185)
CMF	0.415443 (0.0407)	0.432831 (0.0596)	0.752132 (0.0154)	0.731174 (0.0140)
SRCMF	0.378573 (0.0318)	0.589037 (0.0183)	0.774355 (0.0117)	0.746004 (0.0198)
ADA-GRMFC	**0.456657** (0.0393)	**0.598742** (0.0439)	**0.812833** (0.0108)	**0.806995** (0.0098)

The maximum AUPR on each dataset is shown in bold. Standard deviation is shown in parentheses

### Ablation experiments

Our model includes two graph dual regularization terms: a regularization term for drugs and regularization term for targets. To evaluate the impact of the graph dual regularization terms on the performance of ADA-GRMFC, we conduct ablation experiments on the benchmark datasets.

In Tables 6, 7, 8 and 9, parameter $\lambda _d$ and $\lambda _t$ of ADA-GRMFC are chosen using grid search. ADA-GRMFC with $\lambda _d=0$ means that the graph regularization term for drugs is not used. ADA-GRMFC with $\lambda _t=0$ means that the graph regularization terms for targets is not used. When we use regularization terms for drugs and targets, ADA-GRMFC has the highest prediction performance in $CV_d$ and $CV_t$. In $CV_d$, when $\lambda _d=0$, the values of AUC and AUPR of ADA-GRMFC are significantly decreased. The AUC values have decreased by 23%, 19%, 44%, 36% on NR, GPCR, IC and E datasets, respectively. The AUPR values have decreased by 92%, 87%, 91%, 97% on NR, GPCR, IC and E datasets, respectively. Similarly, in $CV_t$, if the graph regularization terms for targets is not used, the performances of ADA-GRMFC is significantly decreased too. When $\lambda _t=0$, the AUC values have decreased by 34%, 33%, 43%, 45% on NR, GPCR, IC and E datasets, respectively. The AUPR values have decreased by 77%, 91%, 95%, 98% on NR, GPCR, IC and E datasets, respectively. These results show that regularization terms for drugs and targets contribute the improvement of DTI prediction performance of ADA-GRMFC significantly.

**Table 6:** AUC results for ADA-GRMFC variants under $CV_d$

Method	NR	GPCR	IC	E
ADA-GRMFC	**0.864387** (0.0153)	**0.826039** (0.0119)	**0.798762** (0.0158)	**0.834382** (0.0082)
ADA-GRMFC$(\lambda _d=0)$	0.668567 (0.0040)	0.668567 (0.0040)	0.450439 (0.0055)	0.534526 (0.0039)
ADA-GRMFC$(\lambda _t=0)$	0.860748 (0.0138)	0.807354(0.0090)	0.756596 (0.0028)	0.785171 (0.0139)

The maximum AUC result in each column is bold. Standard deviation is shown in parentheses

**Table 7:** AUPR results for ADA-GRMFC variants under $CV_d$

Method	NR	GPCR	IC	E
ADA-GRMFC	**0.575141** (0.0388)	**0.381322** (0.0130)	**0.374033** (0.0165)	**0.39878** (0.0112)
ADA-GRMFC$(\lambda _d=0)$	0.0487678 (0.0007)	0.0496542 (0.0007)	0.0346745 (0.0006)	0.0110594 (0.0001)
ADA-GRMFC$(\lambda _t=0)$	0.574956 (0.0200)	0.370817 (0.0058)	0.35606 (0.0122)	0.379671 (0.0168)

The maximum AUPR result in each column is bold. Standard deviation is shown in parentheses

**Table 8:** AUC results for ADA-GRMFC variants under $CV_t$

Method	NR	GPCR	IC	E
ADA-GRMFC	**0.799721** (0.0154)	**0.896419** (0.0245)	**0.948086** (0.0038)	**0.939765** (0.0070)
ADA-GRMFC$(\lambda _d=0)$	0.71424 (0.0342)	0.849599 (0.0186)	0.94421 (0.0013)	0.926141 (0.0074251)
ADA-GRMFC$(\lambda _t=0)$	0.528071 (0.0033)	0.604348 (0.0584)	0.542624 (0.0209)	0.514737 (0.0112512)

The maximum AUC result in each column is bold. Standard deviation is shown in parentheses

**Table 9:** AUPR results for ADA-GRMFC variants under $CV_t$

Method	NR	GPCR	IC	E
ADA-GRMFC	**0.456657** (0.0393)	**0.598742** (0.0439)	**0.812833** (0.0108)	**0.806995** (0.0098)
ADA-GRMFC$(\lambda _d=0)$	0.454528 (0.0240)	0.558464 (0.0287)	0.799589 (0.0087)	0.780136 (0.0157)
ADA-GRMFC$(\lambda _t=0)$	0.105986 (0.0102)	0.0540433 (0.0101)	0.0423862 (0.0026)	0.0114845 (0.0011)

The maximum AUPR result in each column is bold. Standard deviation is shown in parentheses

### Case studies

To further evaluate the ability of ADA-GRMFC to find new targets for a drug and new drugs for a target in practice, two case studies concerning the drug olanzapine and the target estrogen receptor alpha were conducted.

In the first case study, we predicted targets that interact with the drug olanzapine on the G protein-coupled receptors (GPCR) dataset using ADA-GRMFC. Olanzapine is an antipsychotic drug which could target many receptors, and it was recently found that olanzapine could be an attractive antiemetic drug [52]. The known interactions of olanzapine with targets were deleted from the the training dataset, and the candidate targets of olanzapine predicted by ADA-GRMFC were prioritized according to the prediction scores. At last, the top 10 highest-scoring predicted targets were picked out to be validated using the databases KEGG [53] and DrugBank [54]. The results showed that all 10 targets were correctly predicted. The detailed results of the predictions are shown in Table 10.

In the second case study, we predicted candidate drugs for the target estrogen receptor alpha (ER$\alpha$) on the NR dataset and aimed to assess the ability of ADA-GRMFC to predict candidate drugs for targets with no known targeting drugs. ER$\alpha$ is mainly expressed in reproductive tissues (uterus, ovaries), breast, kidney, bone, white adipose tissue and liver, and is over-expressed in more than half of breast cancers [55]. The known interactions of ER$\alpha$ with drugs were removed from the training dataset, and the candidate drugs of ER$\alpha$ predicted by ADA-GRMFC were prioritized according to the prediction scores. The top 20 highest-scoring predicted drugs were selected to be validated against the databases KEGG and DrugBank. Among the predicted 20 drugs, 17 drugs had evidences to target ER$\alpha$. The detailed results of the case study are shown in Table 11.

**Table 10:** Top 10 predicted targets of olanzapine by ADA-GRMFC on the NR dataset

Rank	Name of targets	ID	Evidence
1	5-hydroxytryptamine receptor 2A	**hsa3356**	KEGG &DrugBank
2	adrenoceptor alpha 1A	**hsa148**	KEGG &DrugBank
3	5-hydroxytryptamine receptor 2C	**hsa3358**	KEGG &DrugBank
4	adrenoceptor alpha 2A	**hsa150**	KEGG
5	adrenoceptor alpha 1D	**hsa146**	KEGG
6	dopamine receptor D2	**hsa1813**	KEGG &DrugBank
7	adrenoceptor alpha 2B	**hsa151**	KEGG
8	adrenoceptor alpha 1B	**hsa147**	KEGG &DrugBank
9	5-hydroxytryptamine receptor 1D	**hsa3352**	DrugBank
10	5-hydroxytryptamine receptor 1B	**hsa3351**	DrugBank

Known interactions are in bold

**Table 11:** Top 20 predicted drugs targeting estrogen receptor alpha by ADA-GRMFC on the GPCR dataset

Rank	Name of drugs	ID	Evidence
1	Diethylstilbestrol	**D00577**	KEGG &DrugBank
2	Estramustine	**D04066**	KEGG &DrugBank
3	Estradiol	**D00105**	KEGG &DrugBank
4	Fulvestrant	**D01161**	KEGG &DrugBank
5	Raloxifene hydrochloride	**D02217**	KEGG &DrugBank
6	Desogestrel	**D02367**	KEGG &DrugBank
7	Levonorgestrel	**D00950**	KEGG &DrugBank
8	Norgestrel	**D00954**	KEGG
9	Ethynodiol diacetate	**D01294**	KEGG &DrugBank
10	Progesterone	**D00066**	KEGG &DrugBank
11	Ethinyl estradiol	**D00554**	KEGG &DrugBank
12	Estrone	**D00067**	KEGG &DrugBank
13	Estrone sodium sulfate	**D00312**	KEGG
14	Dienestrol	**D00898**	KEGG &DrugBank
15	Clomiphene citrate	**D00962**	KEGG &DrugBank
16	Fluoxymesterone	**D00327**	KEGG &DrugBank
17	Norethindrone	D00182	unknown
18	Mifepristone	D00585	unknown
19	Medroxyprogesterone acetate	**D00951**	DrugBank
20	Dydrogesterone	D01217	unknown

Known interactions are in bold

## Conclusion

The knowledge of interactions between drugs and targets could help to find the novel usage of drugs. In the paper, we propose a matrix factorization based method, ADA-GRMFC, to predict interactions between drugs and targets. ADA-GRMFC uses graph dual regularization terms to capture structural information from the drug similarity matrix and the target similarity matrix. At the same time, the prior knowledge consistency constraint is used to ensure the matrix decomposition result is consistent with the known DTIs. Finally, an alternating direction algorithm is used to solve the matrix factorization with constraints. Extensive experiments show that ADA-GRMFC outperforms the state-of-the-art methods in predicting DTIs.

The reasons for the superior performance of ADA-GRMFC are as follows. First, unlike traditional matrix factorization algorithms, the prior knowledge consistency constraint ensures that the matrix decomposition result is consistent with the prior knowledge of known DTIs. Second, the graph dual Laplace regular terms not only overcome overfitting of model, but also obtain underlying structural information about the data. Finally, we use alternating direction algorithm with fast convergence to solve the constrained problem.

However, ADA-GRMFC also has limitations. The lack of known drug–target associations may affect the performance of ADA-GRMFC, and including more information related with drugs and targets would help to improve the prediction ability. The values of the parameters of ADA-GRMFC are set by grid search which is time consuming, and appropriate methods to choose optimal parameters need further research.

## Supplementary Information

**Additional file 1.** Convergence of ADA-GRMFC.

## Data Availability

ADA-GRMFC is implemented in Matlab and freely available to the public on https://github.com/zhang340jj/ADAGRMFC/tree/master.
