# Supplementary material for: Graph regularized non-negative matrix factorization with prior knowledge consistency constraint for drug–target interactions prediction
Source: BMC Bioinformatics. 2022 Dec 29;23:564. doi: 10.1186/s12859-022-05119-6 (PMC9798666; doi:10.1186/s12859-022-05119-6)
Supplement: Supplementary file 1 — Additional file 1. Convergence of ADA-GRMFC. [file 12859_2022_5119_MOESM1_ESM.pdf]

Graph regularized non-negative matrix factorization with prior knowledge consistency constraint for drug-target interaction prediction (Appendix)

Junjun Zhang[1] and Minzhu Xie[1,2*]

[1] Key Laboratory of Computing and Stochastic Mathematics(LCSM) (Ministry of Education), School of Mathematics and Statistics, Hunan Normal University, Changsha 410081, China
[2] College of Information Science and Engineering, Hunan Normal University, Changsha 410081, China

Appendix

Convergence

The optimization problem (12) of drug-target interaction prediction is non-convex, and therefore is NP hard. The updating formula (14) could not guarantee to reach a global optimal solution. In this section, we give the proof that ADA-GRMFC could converge to a local optimal solution under some assumptions.

For nonlinear programming, Karush-Kuhn-Tucker (KKT) condition is a necessary condition for a constrained local optimum. For the optimization problem (12), a point (X, Y, M, U, V) satisfies KKT condition if there exist Λ and Π such that

$$\begin{aligned}
&(XY^T - M)Y + \lambda_d \widetilde{\mathcal{L}}_d X + \Lambda = 0,\\
&X^T(XY^T - M) + \lambda_t \widetilde{\mathcal{L}}_t Y + \Pi = 0,\\
&\mathscr{P}_{\Omega^c}(XY^T - M) = 0,\\
&\mathscr{P}_{\Omega}(Z - M) = 0,\\
&X - U = 0,\\
&Y^T - V = 0,\\
&\Lambda \le 0 \le U, \Lambda \odot U = 0,\\
&\Pi \le 0 \le V, \Pi \odot V = 0,
\end{aligned} \tag{1}$$

where Ω^c indexes the unknown drug-target interaction elements of Z, and $\odot$ denotes component-wise multiplication. For brevity, let $W := (X, Y, M, U, V)$ and $\mathscr{L}(X)$ denotes the Lagrangian function with respect to X.

In order to prove local convergence property of the ADA-GRMFC, we first prove the boundness of the graph dual regularization terms. *i.e.* $\left\|\mathrm{Tr}(X^T \widetilde{\mathcal{L}}_d X)\right\| \le n^2$, $\left\|\mathrm{Tr}(Y^T \widetilde{\mathcal{L}}_t Y)\right\| \le m^2$.

$$\begin{aligned}
\left\|X^T \widetilde{\mathcal{L}}_d X\right\| &= \left\|X^T \left(D^d\right)^{-1/2} \mathcal{L}_d \left(D^d\right)^{-1/2} X\right\|\\
&\le \left\|X^T((n-1)^{-1/2}E)((n-1)\,E)((n-1)^{-1/2}E)X\right\|\\
&= \left\|X^T X\right\|\\
&\le \left\|I^T I\right\|
\end{aligned}$$

where E is identity matrix, I denotes all-ones matrix, $E, I \in n \times n$. Then $\left\|\mathrm{Tr}(X^T \widetilde{\mathcal{L}}_d X)\right\| \le \left\|\mathrm{Tr}(I^T I)\right\| = n^2$. Similarly, the same argument can be applied to $\mathrm{Tr}(Y^T \widetilde{\mathcal{L}}_t Y)$, then $\left\|\mathrm{Tr}(Y^T \widetilde{\mathcal{L}}_t Y)\right\| \le m^2$.

Theorem 1. *$\{(W_i, \Lambda_i, \Pi_i)\}$ is the sequence generated by Equation (14). If the sequence of multipliers $\{(\Lambda_i, \Pi_i)\}$ is bounded and satisfies $\sum_{i=0}^{\infty} (\|\Lambda_{i+1} - \Lambda_i\|_F^2 + \|\Pi_{i+1} - \Pi_i\|_F^2) < \infty$. Then any accumulation point of $\{W_i\}$*

satisfies the KKT condition for the (12). Furthermore, we can obtain any accumulation point of $\{X_i, Y_i^T\}$ satisfies the KKT condition of (11).

Proof. In order to make Theorem 1 hold, it is necessary to prove that $W_{i+1} - W_i \to 0$ and $(\Lambda_{i+1}, \Pi_{i+1}) - (\Lambda_i, \Pi_i) \to 0$. $\mathrm{Tr}(X^T \widetilde{\mathcal{L}}_d \mathrm{X})$, $\mathrm{Tr}(Y^T \widetilde{\mathcal{L}}_t Y)$ and (Λ, Π) are bounded. The Lagrangian function $\mathscr{L}(W, \Lambda, \Pi)$ is as follows.

$$\begin{aligned}\mathscr{L}(W, \Lambda, \Pi) =& \frac{1}{2}\left\|M - XY^T\right\|_F^2 + \frac{\alpha}{2}\left\|X - U + \frac{\Lambda}{\alpha}\right\|_F^2 - \frac{1}{2\alpha}\left\|\Lambda\right\|_F^2 \\ &+ \frac{\beta}{2}\left\|Y^T - V + \frac{\Pi}{\beta}\right\|_F^2 - \frac{1}{2\beta}\left\|\Pi\right\|_F^2 + \lambda_d \mathrm{Tr}(X^T \widetilde{\mathcal{L}}_d X) + \lambda_t \mathrm{Tr}(Y^T \widetilde{\mathcal{L}}_t Y),\end{aligned}$$

$\mathscr{L}(W, \Lambda, \Pi)$ is bounded.

In addition, the Lagrangian function $\mathscr{L}$ is strongly convex with respect to each variable of X, Y, M, U, V. It holds for any X and $\Delta\mathrm{X}$ that

$$\mathscr{L}(X + \Delta\mathrm{X}) - \mathscr{L}(X) \geq \partial_X \mathscr{L}(X)^T \Delta\mathrm{X} + \alpha\left\|\Delta\mathrm{X}\right\|_F^2. \tag{2}$$

Furthermore, X^* is the minimum of $\mathscr{L}(X)$, then

$$\partial_X \mathscr{L}(X^*)^T \Delta\mathrm{X} \geq 0. \tag{3}$$

Combining Equation (2) and Equation (3), X_{i+1} is the minimum of $\mathscr{L}(X)$ at the i-th iteration, we have

$$\mathscr{L}(X_i) - \mathscr{L}(X_{i+1}) \geq \alpha\left\|X_i - X_{i+1}\right\|_F^2, \tag{4}$$

and in the same way, we have

$$\mathscr{L}(Y_i) - \mathscr{L}(Y_{i+1}) \geq \beta\left\|Y_i - Y_{i+1}\right\|_F^2, \tag{5}$$

$$\mathscr{L}(M_i) - \mathscr{L}(M_{i+1}) \geq \left\|M_i - M_{i+1}\right\|_F^2, \tag{6}$$

$$\mathscr{L}(U_i) - \mathscr{L}(U_{i+1}) \geq \alpha\left\|U_i - U_{i+1}\right\|_F^2, \tag{7}$$

$$\mathscr{L}(V_i) - \mathscr{L}(V_{i+1}) \geq \beta\left\|V_i - V_{i+1}\right\|_F^2. \tag{8}$$

Let $c := \min\{\alpha, \beta, 1\}$. By Equation (4)-(8), we have

$$\begin{aligned}&\mathscr{L}(W_i, \Lambda_i, \Pi_i) - \mathscr{L}(W_{i+1}, \Lambda_{i+1}, \Pi_{i+1}) \\ &= \mathscr{L}(W_i, \Lambda_i, \Pi_i) - \mathscr{L}(W_{i+1}, \Lambda_i, \Pi_i) \\ &+ \mathscr{L}(W_{i+1}, \Lambda_i, \Pi_i) - \mathscr{L}(W_{i+1}, \Lambda_{i+1}, \Pi_{i+1}) \\ &\geq c\left\|W_i - W_{i+1}\right\|_F^2 - \frac{1}{\gamma\alpha}\left\|\Lambda_i - \Lambda_{i+1}\right\|_F^2 - \frac{1}{\gamma\beta}\left\|\Pi_i - \Pi_{i+1}\right\|_F^2 \\ &\geq c\left\|W_i - W_{i+1}\right\|_F^2 - \frac{1}{\gamma c}(\left\|\Lambda_i - \Lambda_{i+1}\right\|_F^2 + \left\|\Pi_i - \Pi_{i+1}\right\|_F^2).\end{aligned}$$

Summing the above inequalities and $\mathscr{L}(W, \Lambda, \Pi)$ is bounded, we have

$$\sum_{i=0}^{\infty} c\left\|W_i - W_{i+1}\right\|_F^2 - \sum_{i=0}^{\infty} \frac{1}{\gamma c}(\left\|\Lambda_i - \Lambda_{i+1}\right\|_F^2 + \left\|\Pi_i - \Pi_{i+1}\right\|_F^2) < \infty.$$

Since $\sum_{i=0}^{\infty} \frac{1}{\gamma c}(\left\|\Lambda_i - \Lambda_{i+1}\right\|_F^2 + \left\|\Pi_i - \Pi_{i+1}\right\|_F^2)$ is bounded, we have

$$\sum_{k=0}^{\infty} c\left\|W_i - W_{i+1}\right\|_F^2 < \infty,$$

from which we can get $W_{i+1}-W_i \to 0$. Since $\sum_{i=0}^{\infty}(\|\mathit{\Lambda}_{i+1}-\mathit{\Lambda}_i\|_F^2+\|\mathit{\Pi}_{i+1}-\mathit{\Pi}_i\|_F^2) < \infty$ holds, we have $(\mathit{\Lambda}_{i+1},\mathit{\Pi}_{i+1})-(\mathit{\Lambda}_i,\mathit{\Pi}_i)\to 0$.

Next, we prove the result of this theorem. Rearrange the ADA-GRMFC formulas in Equation (14) into

$$(X_{i+1}-X_i)(Y_iY_i^T+\alpha I) = -((X_iY_i-M_i)Y_i^T+\alpha(X_i-U_i)+\mathit{\Lambda}_i+\lambda_d\widetilde{\mathcal{L}}_dX), \tag{9}$$

$$(X_{i+1}^TX_{i+1}+\beta I)(Y_{i+1}-Y_i) = -(X_{i+1}^T(X_{i+1}Y_i-M_i)+\beta(Y_i-V_i)+\mathit{\Pi}_{i+1}+\lambda_t\widetilde{\mathcal{L}}_tY), \tag{10}$$

$$U_{i+1}-U_i = \mathscr{P}_+(X_{i+1}-\frac{\mathit{\Lambda}_i}{\alpha})-U_i, \tag{11}$$

$$V_{i+1}-V_i = \mathscr{P}_+(Y_{i+1}-\frac{\mathit{\Pi}_i}{\beta})-V_i, \tag{12}$$

$$\mathit{\Lambda}_{i+1}-\mathit{\Lambda}_i = \gamma\alpha(X_{i+1}-U_{i+1}), \tag{13}$$

$$\mathit{\Pi}_{i+1}-\mathit{\Pi}_i = \gamma\beta(Y_{i+1}-V_{i+1}), \tag{14}$$

$$M_{i+1} = X_{i+1}Y_{i+1}^T-\mathscr{P}_\Omega(Z-X_{i+1}Y_{i+1}^T). \tag{15}$$

Note $W_{i+1}-W_i\to 0$, $\mathit{\Lambda}_{i+1}-\mathit{\Lambda}_i\to 0$ and $\mathit{\Pi}_{i+1}-\mathit{\Pi}_i\to 0$ imply that both the left-hand and right-hand sides of Equation (9)-(15) converge to zero, *i.e.*,

$$(X_iY_i-M_i)Y_i^T+\mathit{\Lambda}_i+\lambda_d\widetilde{\mathcal{L}}_dX\to 0, \tag{16}$$

$$X_i^T(X_iY_i-M_i)+\mathit{\Pi}_{i+1}+\lambda_t\widetilde{\mathcal{L}}_tY\to 0, \tag{17}$$

$$\mathscr{P}_+(X_{i+1}+\frac{\mathit{\Lambda}_i}{\alpha})-U_i\to 0, \tag{18}$$

$$\mathscr{P}_+(Y_{i+1}+\frac{\mathit{\Pi}_i}{\beta})-V_i\to 0, \tag{19}$$

$$X_i-U_i\to 0, \tag{20}$$

$$Y_i-V_i\to 0, \tag{21}$$

the terms $\alpha(X_i-U_i)$ and $\beta(Y_i-V_i)$ in Equation (16) and Equation (17) have been eliminated by Equation (20) and Equation (21), respectively. For any limit point $\hat{W}=(\hat{X},\hat{Y},\hat{M},\hat{U},\hat{V})$ of the sequence $\{W_i\}$, there exists subsequence $\{W_{n_i}\}$ converging to $\hat{W}$. Since $\{(\mathit{\Lambda}_i,\mathit{\Pi}_i)\}$ is bounded, there exists a subsequence $\{(\mathit{\Lambda}_{n_{i_i}},\mathit{\Pi}_{n_{ij}})\}$ of $\{(\mathit{\Lambda}_{n_i},\mathit{\Pi}_{n_i})\}$ that converges to $(\hat{\mathit{\Lambda}},\hat{\mathit{\Pi}})$. Hence $(\hat{W},\hat{\mathit{\Lambda}},\hat{\mathit{\Pi}})$ is a limit point of the sequence $(W_i,\mathit{\Lambda}_i,\mathit{\Pi}_i)$. From Equation (15), we have

$\mathscr{P}_\Omega(M_i-Z)=0$, $\mathscr{P}_\Omega(X_iY_i{}^T-M_i)=0$.

The limit point $(\hat{W},\hat{\mathit{\Lambda}},\hat{\mathit{\Pi}})$ satisfies the first six equations in Equation (1). The nonnegativity of $\hat{U}$ and $\hat{V}$ can be guaranteed by the ADA-GRMFC construction. Therefore, the nonnegativity of $\hat{\mathit{\Lambda}}$ and $\hat{\mathit{\Pi}}$ and the complementarity between $\hat{U}$ and $\hat{\mathit{\Lambda}}$, and between $\hat{V}$ and $\hat{\mathit{\Pi}}$ need to be proved. The following two equations can be obtained from Equation (18) and Equation (19):

$$\mathscr{P}_+(\hat{X}+\frac{\hat{\mathit{\Lambda}}}{\alpha})=\hat{U} \tag{22}$$

$$\mathscr{P}_+(\hat{Y}+\frac{\hat{\mathit{\Pi}}}{\beta})=\hat{V} \tag{23}$$

Note we have $\hat{X}=\hat{U}\geq 0$. If $\hat{U}_{ij}=\hat{X}_{ij}=0$, then $\mathscr{P}_+(\frac{\hat{\mathit{\Lambda}}}{\alpha})_{ij}=0$, $\hat{\mathit{\Lambda}}_{ij}\leq 0$. On the other hand, if $\hat{U}_{ij}=\hat{X}_{ij}>0$, we have $\hat{\mathit{\Lambda}}_{ij}=0$. Thus the non-positivity of $\hat{\mathit{\Lambda}}$ and the complementarity between U and $\hat{\mathit{\Lambda}}$ are proved. Similarly, the same argument can be applied to Equation (23), the non-positivity of $\hat{\mathit{\Pi}}$ and the complementarity between $\hat{V}$ and $\hat{\mathit{\Pi}}$ can be proved.

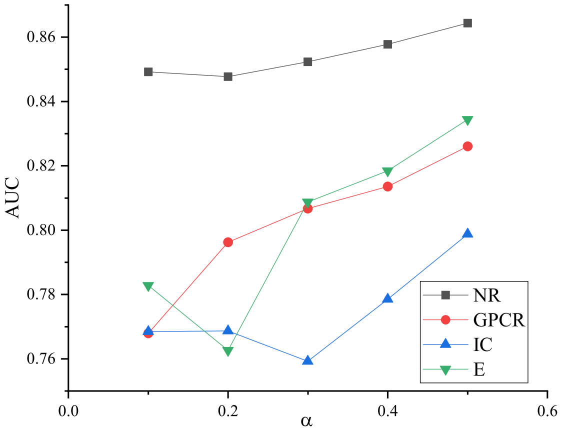

(a) CV_d

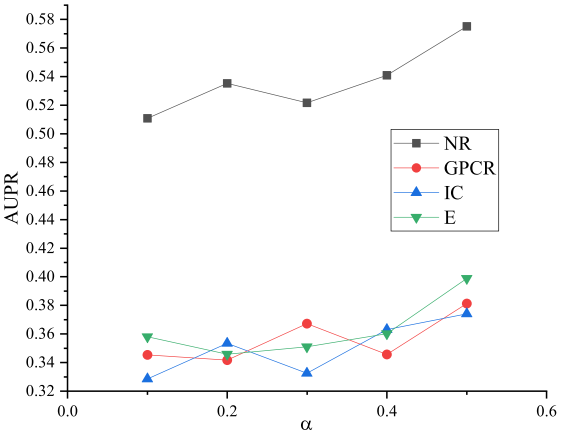

(b) CV_d

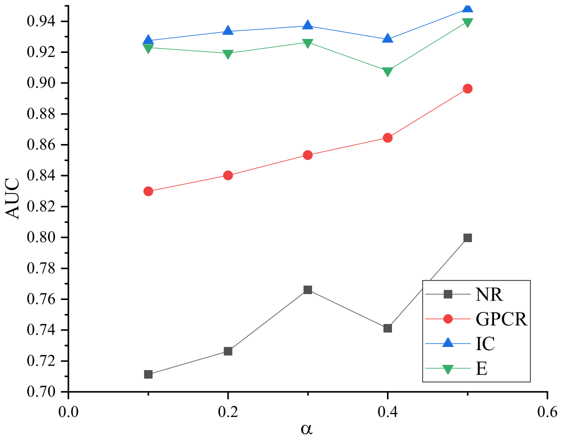

(c) CV_t

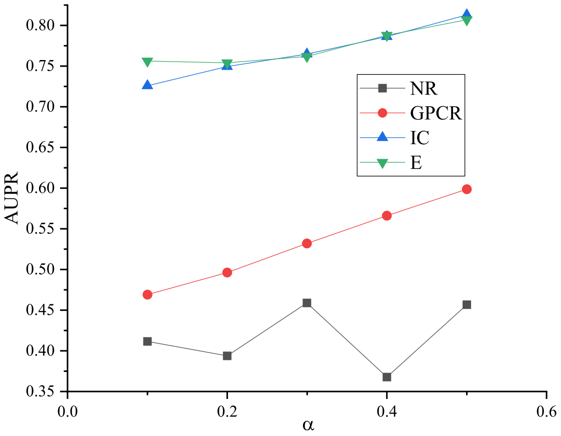

(d) CV_t

Fig. 1: AUC and AUPR of ADA-GRMFC for different α under CV_d and CV_t, where $\beta = 0.01$ and $\gamma = 1.618$.

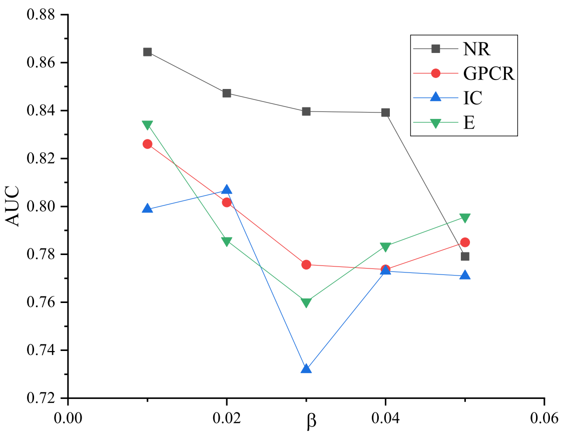

(a) CV_d

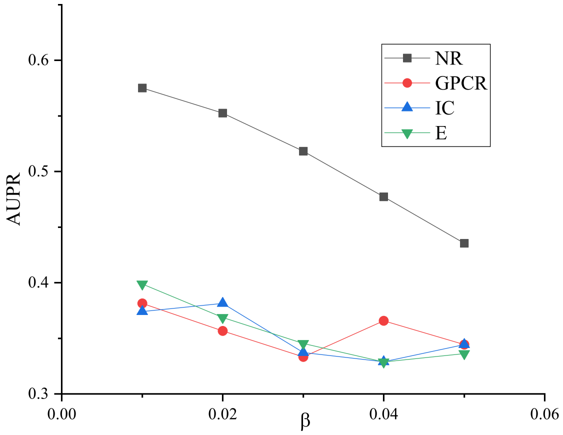

(b) CV_d

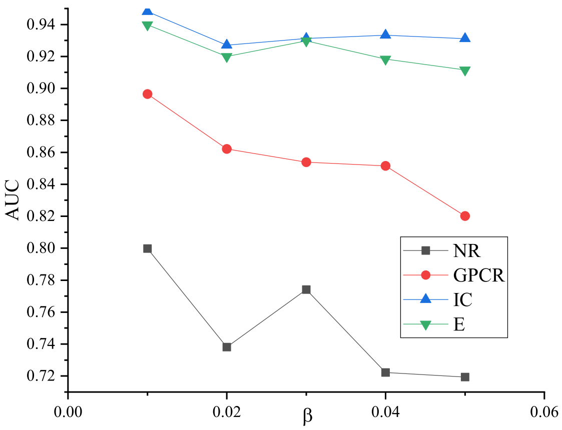

(c) CV_t

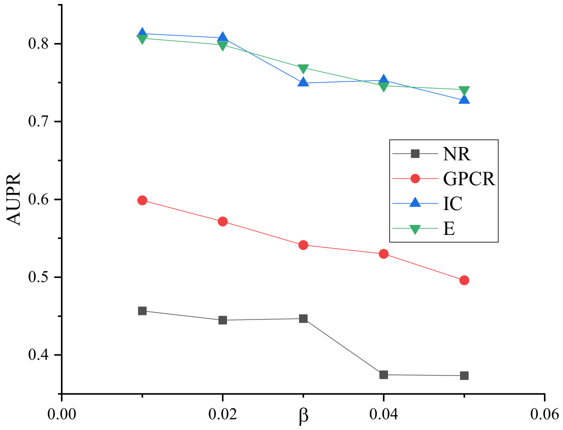

(d) CV_t

Fig. 2: AUC and AUPR of ADA-GRMFC for different β under CV_d and CV_t, where $\alpha = 0.5$, $\gamma = 1.618$.

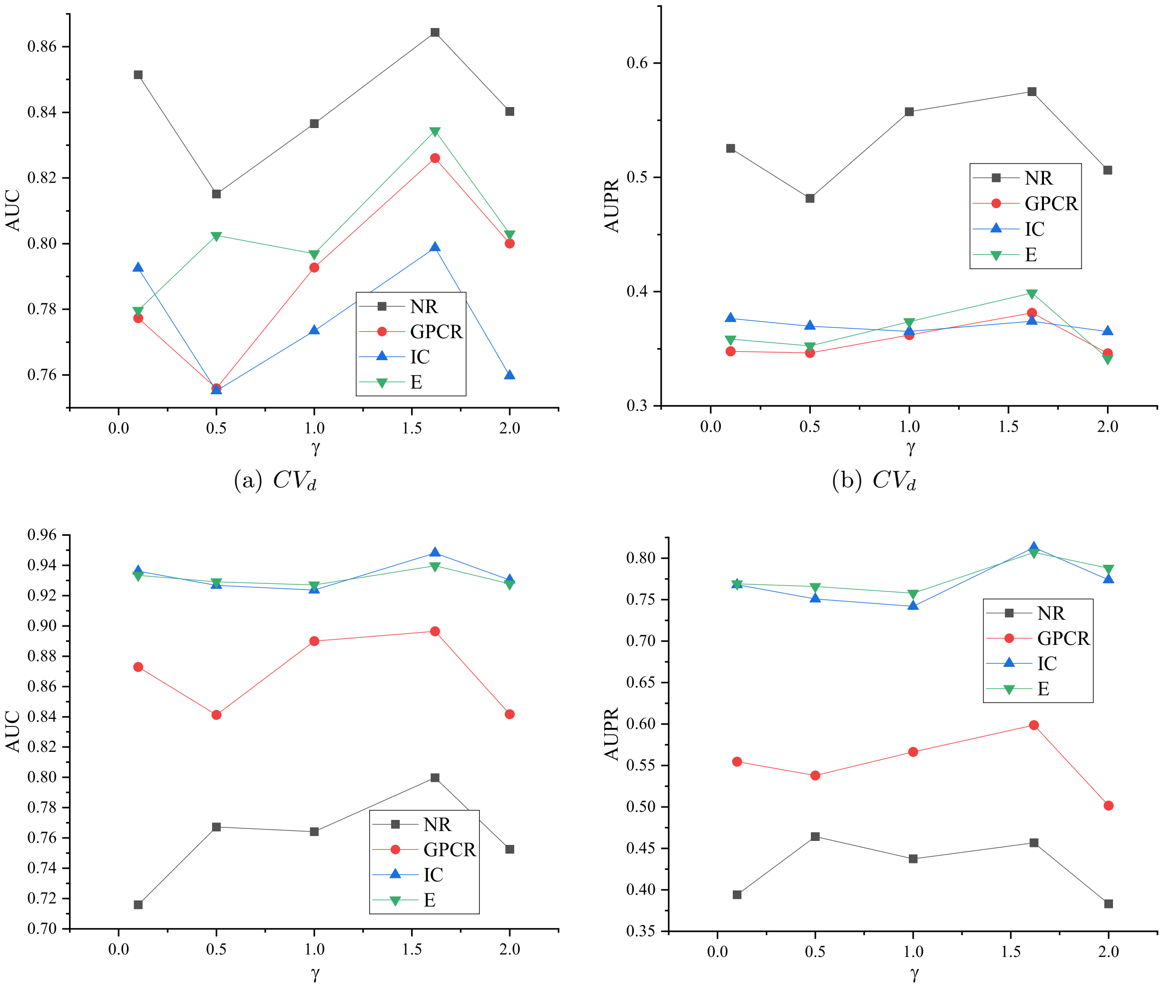

(a) CV_d (b) CV_d

(c) CV_t (d) CV_t

Fig. 3: AUC and AUPR of ADA-GRMFC for different γ under CV_d and CV_t, where $\alpha = 0.5$, $\beta = 0.01$.
